# Grand strategy and the construction of the national interest: the underpinnings of Sino-US strategic competition

**DOI:** 10.1057/s41311-023-00452-w

**Published:** 2023-04-26

**Authors:** Sabine Mokry

**Affiliations:** 1 German Institute for Global and Area Studies (GIGA), Rothenbaumchaussee 32, 20148 Hamburg, Germany; 2 Institute of Political Science, Leiden University, Leiden, The Netherlands

**Keywords:** Grand strategy, National interest, US foreign policy, Chinese foreign policy, Great power competition, Constructivism

## Abstract

The ubiquity of Sino-US strategic competition calls for uncovering its underpinnings by systematically comparing their grand strategies. This article examines how both governments have constructed their countries’ national interest since the 2008 global financial crisis. Through quantitative content analysis, it traces the relative salience of different components of the construction of the national interest detailing differences and shifts in emphasis and divergences within both governments. Whereas the Chinese government increasingly emphasized taking on a leadership role in international affairs, the US government became more concerned about the country’s security and economic standing. Between Chinese actors, the most pronounced divergence appeared regarding the importance of territorial defense. US actors disagreed about the importance of promoting the country’s values abroad. These findings suggest that a long-term perspective is needed to uncover the underpinnings of great power competition. Researchers and policy-makers must listen to different foreign policy actors, even under centralized authoritarian rule.

## Introduction

The intensifying strategic competition between the USA and the People’s Republic of China (PRC) permeates almost all aspects of world politics. It affects, for instance, how new technological infrastructure is regulated, how global development is envisioned, and how the world responds to global health emergencies, such as the COVID-19 pandemic. The “return of great power politics” has been described as the “defining feature of the twenty-first century” (Norrlöff 2021, p. 7). Since 2018, the US government has referred to China and Russia as “strategic competitors” (USA 2018). Despite Russia attacking another sovereign country in February 2022, US Secretary of State Anthony Blinken cast China as the more formidable rival over the long term. He described the PRC as “the only country with the intention to reshape the international order and, increasingly, the economic, diplomatic, military, and technological power to do it” (Blinken 2022). So far, International Relations scholars have examined Sino-US strategic competition in different fields, from the financial sector (Zhang 2020) to digital politics (Yan 2020), reflected upon implications for third parties (Lobell 2011; Han 2018; Cha 2020; She 2021), and expressed growing concerns about the potential of escalation and a “new Cold War” (Yan and Qi 2012; Layne 2020; Zhang and Xu 2021). To grasp the underpinnings of Sino-US great power competition with its far-reaching implications for the bilateral relationship and international affairs more broadly, this article systematically analyzes a broad sample of Chinese and US foreign policy statements stretching across different policy-making levels and covering different foreign policy actors within both governments.

This article contributes to the burgeoning scholarship that examines grand strategy from a constructivist perspective. Conceiving grand strategy as constructed allows us to account for changes over time and uncover divergences within each state. Both tend to be hidden in realist accounts of grand strategy that dominate the debate (Löfflmann 2020, p. 589). This article zooms in on the starting point of any grand strategy: the national interest (Goddard 2021, p. 324). Understanding the national interest as constructed, it distinguishes six components of the construction of the national interest: First, *defend the country’s territory, political system, and citizens*, second, *expand the country’s economic relations*, third, *lead global governance*, fourth, *offer global public goods*, fifth, *control the region*, and sixth, *promote the country’s values*. Empirically, the article compares the underpinnings of great power competition in the USA and China. By starting its analysis with the global financial crisis in 2008, it provides a longer-term perspective on shifts in US and Chinese grand strategy stretching across different administrations.

Through quantitative computer-assisted content analysis, this article compares official foreign policy statements from the USA and China, including strategic guidelines, policy papers, and speeches in front of international and domestic audiences. Inspired by Robertson (2017), the article distinguishes the strategic from the policy planning level (2017). Outlining broad and long-term directions, foreign policy statements at the strategic level are the most authoritative. In the Chinese context, this applies to the Political Work Report to Party Congress and other statements by the Chinese Communist Party (CCP) General Secretary/State President. In the USA, this applies to statements from the President and the National Security Strategy. Statements on the policy planning level describe more focused communication related to a particular context. This level covers speeches by ministers/cabinet members tasked with foreign policy issues and policy papers on specific issues. In the Chinese context, the policy planning level covers speeches by the Premier and Foreign Minister and policy papers issued by the State Council Information Office (SCIO).^1^ In the USA, this level covers the Vice-President and the Secretaries of State and Defense.^2^ The analysis starts right after the global financial crisis in 2008 and ends after the first waves of the COVID-19 pandemic in June 2022.^3^

The analysis reveals important differences in emphasis between the USA and China, significant changes over time, and pronounced discrepancies between policy levels and foreign policy actors. Most importantly, the Chinese government seeks to take on a leadership role in international politics, while the US government becomes more concerned with the country’s security and economic standing. The ideological confrontation did not play out openly in the analyzed time frame. However, *promote China’s values* became already more important after Xi took office, and since 2020 the relative salience of this component of the national interest has also become more important on the US side. In many instances, the manifestations of the USA and Chinese grand strategy, for example, regarding their positions in the Asia–Pacific region, mirror each other. There were more divergences between policy levels in the USA than in Chinese foreign policy statements. However, even in the PRC’s highly centralized authoritarian system, there were still discrepancies between the strategic and the policy planning level. In the USA, such discrepancies were more frequent under Trump than his predecessor and successors. On the Chinese side, differences were smallest between the CCP General Secretary/State President and other actors. In the USA, this was the case for the State Department and other actors. In the USA, most disagreements between foreign policy actors appeared regarding promoting the country’s values abroad; in China, most disagreements featured regarding the importance of the country’s defense.

The article proceeds as follows: The next section describes grand strategy from a constructivist perspective and introduces the article’s conceptualization of the construction of the national interest. Before presenting the article’s findings, including differences in emphasis in the construction of the national interest between China and the USA, changes in the relative salience of the different components of the construction of the national interest in countries, as well as differences and shifts in the degree of divergence between policy-making levels and between actors within each state, the data, and methods the analysis draws upon are introduced as well as the analysis’ empirical context. What these findings reveal about US and Chinese grand strategies and what they imply for great power competition is also discussed.

## Conceptual framework

One must examine how both countries formulate their grand strategies to grasp the underpinnings of Sino-US strategic competition. This article concentrates on the starting point of any grand strategy: the national interest. It systematically compares the US and the PRC’s constructions of the national interest. After providing a brief overview of the study of grand strategy in the USA and China, the next section introduces key aspects of grand strategy from a constructivist perspective. It thereby highlights the advantages of understanding the national interest as being constructed for uncovering the underpinnings of Sino-US strategic competition and how a new conceptualization of the construction of the national interest allows doing this.

The scholarly attention attributed to US grand strategy far outweighs the attention given to Chinese grand strategy. However, against the backdrop of broader changes in Chinese foreign policy, more scholars have started piecing together China’s grand strategy. Silove’s (2018) distinction into grand behavior, that is, patterns in state behavior, grand principles, which is an “organizing principle that is consciously held and used by individuals to guide their decisions, and grand plans, which is “deliberate, detailed plan devised by individuals” (2018, p. 29) helps structure the debate. Most contributions about Chinese grand strategy examine either patterns in state behavior (Swaine 2000; Fallon 2015; Wang 2016; Mendez and Alden 2021) or “grand principles” (Wang 2011; Zhang 2012; Buzan 2014; Yongnian and Fook 2016; Goldstein 2020; Demirduzen and Thies 2022). Despite being highly necessary, assessments that consider both meanings of grand strategy are still rare (Roy 2022). In addition, hardly any examinations of “grand plans” can be found (Scobell 2022). A similar picture emerges for the USA: Many articles focus on either “grand principles” (Biegon 2019; Kurthen 2021; Janusch 2022) or “grand behavior” (Gregg 2010; Dombrowski and Reich 2017; Hazelton 2017; Desmaele 2021), “grand plans” receive a lot less attention (Drezner 2011; Tabachnik and Miller 2021). In addition, most scholarship on Chinese and US grand strategy conceives of the states as unitary actors. Whereas at least a few scholars try to differentiate between foreign policy actors in the USA (Layne 2017; Löfflmann 2020), divergences between Chinese foreign policy actors remain uncovered.

Beyond the study of individual state’s grand strategies, Goddard and Krebs (2015) observe that “grand strategy is an arena in which realism, rationalism, and materialism have long held sway” (2015:6). This predominance, however, is increasingly challenged by scholars who bring constructivism to the debate about grand strategy. Goddard (2021) draws attention to the role of rhetoric and calls for “treating legitimation as central to the study of grand strategy” because it “drives grand strategy at every state, from the articulation of national interest to the interpretation of threat to the selection of instruments” (2021:322). Similarly, Kornprobst and Traistaru (2021) see grand strategy as interwoven with communication and advocate for using linguistics to study grand strategy (Kornprobst and Traistaru 2021). Other constructivist scholars highlight culture and identity as essential components of grand strategy (McCourt 2021, p. 303). Common to all these approaches is that they “give lie to the rationalist assumption that grand strategy is generated by a unitary actor” (Balzacq et al. 2021:159).

This article builds on recent advances in the study of grand strategy from a constructivist perspective. Goddard and Krebs (2015) distinguish four constituent elements of grand strategy: the articulation of the national interest, the identification and prioritization of threats to the state, the development of ways to address those threats, and the execution of grand strategy by mobilizing necessary resources at home and abroad (2015, p. 8). This article zooms in on the first element of grand strategy, the “well-defined national interest” […] that “specifies what ends a collective actor, usually the state, should pursue in world politics” (Goddard 2021, p. 324). In contrast to Goddard and her focus on legitimation, which shows why the construction of the national interest matters, the approach introduced here allows us to uncover *how* the national interest is constructed. The benefit of examining how the national interest is constructed lies in comparing the grand strategies of different states with each other and looking inside each state by looking at different policy-making levels and foreign policy actors. By distinguishing different components of the construction of the national interest and tracing shifts in the relative salience of these components over time, the approach allows for bringing in the content of the national interest.

To identify the different components of a country’s national interest, this article links the basic needs Nuechterlein describes in his conception of the national interest to more recent empirical investigations into national interests. First, defending its territory, political system, and citizens are in a country's interest. Nuechterlein describes “defence interests” as “the protection of the nation-state and its citizens against the threat of physical violence directed from another state and/or externally inspired threat to its system of government” (Nuechterlein 1976, p. 248). Second, expanding its external economic relations is in a country’s interest. Nuechterlein describes economic interests as “the enhancement of the nation-state’s well-being in relations with other states” (Nuechterlein 1976, p. 248). The empirical literature on national interests references economic interests: Roberts points to promoting trade (Roberts 2014). Kitaoka refers to the people’s prosperity and identifies free trade as a precondition (Kitaoka 2016, p. 36). Third, it is in a country’s interest to lead global governance. Under “world order,” Nuechterlein discusses the “maintenance of a political and economic system in which the nation-state may feel secure, and its citizens and commerce may operate peacefully outside its borders” (Nuechterlein 1976, p. 248). Fourth, it is in a country’s interest to promote its values. For Nuechterlein, ideological interests refer to “the protection and furtherance of a set of values which the people of a nation-state share and believe to be universally good” (Nuechterlein 1976, p. 248). Reviews of the literature on China’s foreign policy, in particular, suggest that Nuechterlein overlooks regional order and the provision of global public goods. Fifth, it is in a country’s interest to establish itself as the leading power in the region it is situated in. Sixth, providing global public goods is in a country’s interest. Table 1 provides an overview of the components of the construction of the national interest as they apply to great powers.

**Table 1:** Overview of the components of great powers’ constructions of the national interest

Component of the construction of the national interest	Abbreviation	Brief description	Components
Protect the country’s territory, political system, and citizens from external threats	Def	It is in the country’s interest to defend its territory, political system, and citizens from external threats	Defense of the political system Defense of territory Protection of citizens Threats Ways to guarantee security
Expand the country’s external economic relations	Econ	It is in the country’s interest to expand its external economic relations	Domestic economic development International economic cooperation World economic context
Lead global governance	Gov	It is in the country’s interest to lead global governance	Aims Disposition for leadership International context Issue areas
Promote the country’s values	Val	It is in the country’s interest to promote its values	Democratic values & human rights Implementation Outcome Requirements
Control the region	Reg	It is in the country’s interest to establish itself as the leading power in the Asia–Pacific	Aims for the region Forms of regional cooperation Regional context
Offer global public goods	Publ	It is in the country’s interest to provide global public goods	Ends Means

## Methodology

Understanding the national interest as constructed and breaking it down into the six components introduced above that can be traced in foreign policy statements allows us to examine the amount of attention a government attributes to the different components over time. Since the national interest provides the starting point of any grand strategy, such an examination offers important insights into how the country’s grand strategy evolves as it reflects shifts in the ranking of priorities. Distinguishing these six components of the construction of the national interest further allows for examining patterns in relative salience for different policy-making levels and foreign policy actors within each state. Hence, the analysis provides important insights into how a country’s grand strategy evolves while being constructed by foreign policy elites. More concretely, differences in prominence of the different components of the construction of the national interest reveal the making of trade-offs at the heart of grand strategy.

Frame analysis inspired the quantitative content analysis this article is based on. It describes the systematic identification and examination of frames. As schemata of interpretation (Goffman 1974, p. 21), frames are “cognitive tool[s] that help actors organize information in a complex environment” (Lenz 2018, p. 32). Frames are generated by actors who engage in framing processes. Entman (2007) describes framing as a “process of culling a few elements of a perceived reality and assembling a narrative that highlights connections among them to promote a particular interpretation” (2007, p. 164). Codes to identify the frames were developed inductively on the material.^4^ After identifying the frames, they were clustered into themes. As illustrated in Table 1, each of the six components of the construction of the national interest conceptualized above comprises several of these themes. To assess differences in the relative salience of the components of the construction of the national interest, one compares how frequently the different components appear relative to each other. To examine patterns in relative salience over time across policy levels, or foreign policy actors, one calculates the percentages of frames that pertain to each component per year and compares the percentages with each other.

This article remains solely focused on the rhetorical level of grand strategy. Two important limitations come with this focus. First, there might be big differences between what is publicly communicated and what is discussed behind closed doors, not only in a highly secretive authoritarian system such as the PRC (Barros 2016). Second, discrepancies between a state’s communicated grand strategy and behavior may exist. Most recently, Nathan and Zhang (2022), for instance, argued that “Chinese foreign policy behavior often diverges from the face meaning of its rhetoric […]” (2022, p. 58). China’s approach to territorial disputes in the South China Sea is a prominent example of these differences. In its rhetoric, the Chinese government emphasizes that disputes should be settled through mutual respect and negotiations. However, it does not acknowledge the Permanent Court of Arbitration’s ruling and continues building large, militarily fortified artificial islands to stake its claims (ibid, p. 70). This article cannot account for such gaps between foreign policy rhetoric and behavior. However, longer-term analyses of official foreign policy statements can serve as a jumping-off point for identifying such gaps. At the same time, it is still worth paying close attention to foreign policy rhetoric, especially if one analyses it carefully and finds discrepancies between different actors.

## Empirical context

This section briefly outlines the evolution of Sino-US relations during the analyzed time frame to situate the subsequent analysis in its empirical context. In 2008, China became the largest holder of US treasuries (Reuters Staff 2010). Three years later, it became the world’s second-largest economy (*BBC *News 2011). In response to China’s growing economic and political clout, the Obama administration announced its “Pivot to Asia” in 2011. Secretary of State Hillary Clinton called for increased investment to counter China’s growing economic clout (Clinton 2011). Shortly after Xi Jinping took office, Barack Obama hosted him at the Sunnylands Summit to build personal rapport amidst growing tensions (Economy 2013). However, tensions between the two countries continued to build and evolved into a trade war under the Trump administration from 2018 onwards, accompanied by increasingly derogatory rhetoric (Bown and Kolb 2018). While the Biden administration eased its rhetoric slightly, many Trump policies remained in place (Khalid 2021).

When zooming out of specific policies toward the larger strategic context, observers point to fundamental shifts in Sino-US relations (Foot and King 2019; Medeiros 2019). Medeiros (2019), for instance, describes a “unique and worrisome convergence” in both the “longer-term structural drivers” and the “shorter-term cyclical ones” in the bilateral relationship. He sees all drivers push toward a more competitive direction. In addition, he points out that the “classic buffers and stabilizers,” for example, the US business community’s support, are being diminished, if not already inoperative (2019, p. 113). Foot and King share this assessment and add that the close economic relationship that used to be the foundation of USA—China relations is increasingly seen as a problem (2019, p. 48). Yang (2020) points out that while much of the world’s attention has focused on the trade war, there are many more intensifying disputes regarding “China’s industrial policies, ethnic and peripheral policies and overseas ventures, including high technology, regional security, Taiwan and, increasingly, Xinjiang and Hong Kong as well” (Yang 2020, p. 419).

In both states, foreign policy actors are located at different policy-making levels. At the strategic level, the CCP General Secretary/PRC State President and the US President outline the broad and long-term directions of the country’s foreign policy in their statements. In the PRC, in particular, there has been a centralization of political power under Xi Jinping in recent years resulting in Xi Jinping himself and party bodies increasing their control over foreign policy (Lee 2017; Cabestan 2020). The policy planning level, where more focused communication about particular issues is located, is constituted by cabinet members tasked with foreign policy in the USA. In the Chinese context, this level covers the Foreign Minister and the State Council Information Office that is closely linked to the Propaganda Department (Brady 2008) and publishes white papers explaining the Chinese government’s positions on various foreign policy issues to the outside world.

## Findings and implications for grand strategy

This section presents how the US and Chinese governments constructed their countries’ respective national interest between 2008 and 2022. It first offers an overview of differences in the relative salience of the components of the construction of the national interest in US and Chinese foreign policy statements, then traces changes in emphasis over time for both countries, and, ultimately, zooms in on divergences between policy-making levels and foreign policy actors within each state to assess how coherent the construction of the national interest is on both sides.

### Differences in emphasis in the construction of the national interest

**Fig. 1:**
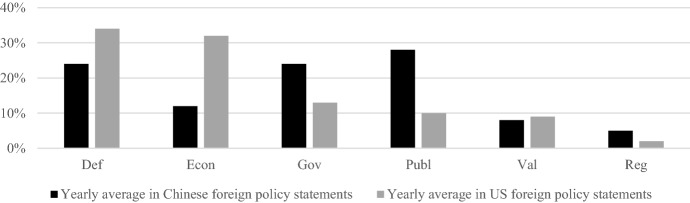
Relative salience of the different components of the construction of the national interest in the US and Chinese foreign policy statements issued between 2008 and 2022

The Chinese and US governments emphasize different aspects when they construct their countries’ national interest. Most importantly, the US government emphasizes the components of the national interest *defend the territory, political system, and citizens* and *expand economic relations*, while the Chinese government puts much more emphasis on *offer global public goods* and *lead global governance,* as Fig. 1 illustrates*.* Both governments put roughly the same limited emphasis on *promote the country’s values*. The Chinese government emphasizes *control the region* slightly more than the US government. For both, it is the least prominent component of the construction of the national interest.

From the relative salience of the different components of the construction of the national interest in the US and Chinese foreign policy statements, several inferences about the countries’ grand strategies and Sino-US great power competition can be drawn. From the Chinese government’s focus on *offer global public goods* and *lead global governance,* one can infer that it seeks to play a more prominent role internationally. The USA, in contrast, voices much less pronounced ambitions in these areas, either because it is already considered the hegemon or because its ambitions for global leadership dwindled. The US government’s increasing concerns about its security and economic standing, visible through its focus on *defend its territory, political system, and citizens*, and *promote its external economic relations*, support the latter assessment.^5^ Both governments put a similar emphasis on promoting the country’s values internationally. Even more importantly, the ambitions expressed in this area pale in comparison with the other components of the national interest. Hence, the ideological confrontation did not play out openly in the analyzed time frame.

### Shifts in emphasis over time in the construction of the national interest

**Fig. 2:**
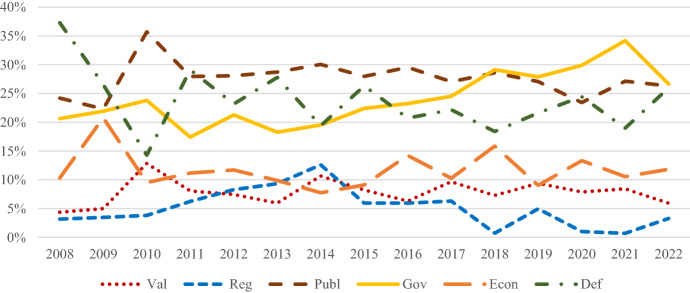
Relative salience of the different components of the construction of China’s national interest over time

**Fig. 3:**
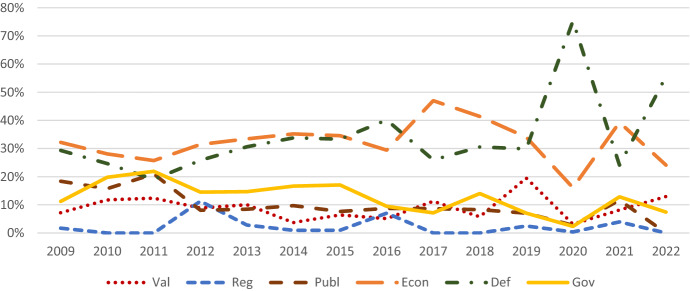
Relative salience of the different components of the construction of China’s national interest over time

Over time, there were significant differences in how much emphasis the Chinese and US government put on the different components of the construction of the national interest, as illustrated by Figs. 2 and 3. In Chinese foreign policy statements, *lead global governance* became more important. While it quickly gained and lost importance between 2008 and 2011, after 2011, its relative salience increased constantly. In 2018, it became the most salient component of the construction of China’s national interest, replacing *offer global public goods*. *Control the region* was mostly the least salient component of the construction of China’s national interest, except for a short-lived peak in emphasis between 2012 and 2014, when it was more important than *expand China’s economic relations* and *promote China’s values*. *Expand China’s economic relations* became more important over time, albeit with frequent shifts in its relative salience. *Promote China’s values* was mostly the second least salient component of the construction of the national interest. A drop immediately followed a brief peak in emphasis in 2010. After 2013, it gained prominence again. There were frequent shifts in the importance attributed to *defend China’s territory, political system, and citizens*. The Chinese government’s increased emphasis on *lead global governance* suggests it seeks to take on a leadership role in international politics. After an initial push for leadership in the region, the Chinese government quickly toned down these ambitions. After Xi Jinping took office, ideology became slightly more important, but its overall importance remains limited.

In contrast to the patterns observed in the Chinese government’s pronouncements, *lead global governance* and *offer global public goods* constantly lost importance in US foreign policy statements. Usually, they ranked as the third and fourth most salient component. Instead, the US government put much more emphasis on *defend its territory, political system, and citizens* and on *expand its economic relations*. The former became particularly salient between 2019 and 2021. *Control the region* was, for the most part, the least salient component of the construction of the national interest. However, there were spikes in emphasis between 2011 and 2013 and 2015 and 2017. Throughout the analyzed time frame, *promote the country’s values* was the second least salient construction of the US national interest. However, the first spike in emphasis in 2017 was followed by another even more pronounced one in 2019. After 2020, *promote US values* became more important while still modest compared to the other components of the construction of the national interest. Notably, this trend started under the Trump administration and continued under Biden. The US government focused less on leading international institutions. This trend was only somewhat reversed at the beginning of the Biden administration.

In direct comparison, shifts in relative salience in the components of the construction of the national interest *lead global governance* and *control the region* in Chinese and US foreign policy statements were most interesting. Between 2010 and 2012, the US and Chinese governments paid roughly the same attention to *lead global governance*. Afterward, the Chinese government constantly increased its emphasis, while the US government’s focus on this component decreased. Regarding *control the region*, between 2011 and 2013, there was a spike in emphasis in US foreign policy statements. This spike was immediately followed by a peak in interest in Chinese foreign policy statements. Between 2015 and 2017 and 2018 and 2020, the pattern repeated itself, albeit to a slightly lesser degree. The more pronounced peak in emphasis in Chinese foreign policy statements between 2018 and 2020 was mirrored between 2020 and 2022 in US foreign policy statements. Hence, the emphasis both governments attach to the Asia–Pacific is heavily influenced by the emphasis the other government attributes to the region.

The above analysis suggests changes in priorities regarding both countries’ leadership roles in international affairs. While the Chinese government constantly emphasized leading global governance and offering global public goods, the US government focused more on expanding its economic relations and defending its territory, political system, and citizens, suggesting an inward turn. The observed patterns in relative salience regarding both countries' roles in the region suggest that both countries’ grand strategies do not develop in a vacuum but mirror each other. Lastly, the frequently involved rivalry between political systems and associated values slowly started to be reflected in US and Chinese foreign policy statements, albeit more pronounced on the US side.

### The divergence between policy-making levels

**Fig. 4:**
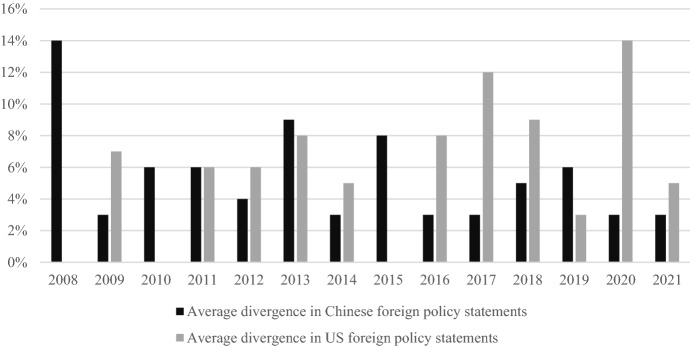
Degree of divergence between policy-making levels in Chinese and US foreign policy statements

In both countries, there was a substantial divergence between foreign policy statements issued at the strategic and policy planning levels, as Fig. 4 shows. In Chinese foreign policy statements, the highest degree of divergence between the strategic and the policy planning level manifested in 2008. Afterward, there was a rapid decrease in divergence. The divergence between policy levels was very small in 2009, 2014, between 2016 and 2017 and between 2020 and 2021. In US foreign policy statements, the highest divergence appeared in 2020. Between 2011 and 2014, the degree of divergence between policy-making levels was fairly constant and at a similar level as in Chinese foreign policy statements. Between 2016 and 2021, there were pronounced shifts in the degree of divergence: In 2017 and 2020, the degree of divergence was fairly high; in 2019 and 2021, it was comparatively low. In direct comparison, the overall degree of divergence between policy-making levels was higher in the USA than in Chinese statements. For both countries, the degree of divergence between policy-making levels fluctuated with no clear trends emerging.

It is hardly surprising that there was more divergence between policy levels in the US democratic system compared to the Chinese authoritarian system. However, differences between the two countries were not as big as expected: Even in the PRC’s highly centralized authoritarian system, discrepancies between the strategic and the policy planning level were uncovered. When Xi Jinping took power, these differences were particularly pronounced. Under his rule, the divergence between policy-making levels decreased, albeit not constantly. In 2018 and 2019, for instance, there was again significant divergence between the strategic and policy planning level. In the USA, the degree of divergence between policy-making levels coincided with the different administrations. During the Trump administration, there were more differences between the strategic and policy planning level than under Obama or Biden. The identified divergences between policy levels hint at inconsistencies in great powers’ grand strategies. The next section will investigate these discrepancies by examining divergences between foreign policy actors within both governments.

### The divergence between actors within the state

**Fig. 5:**
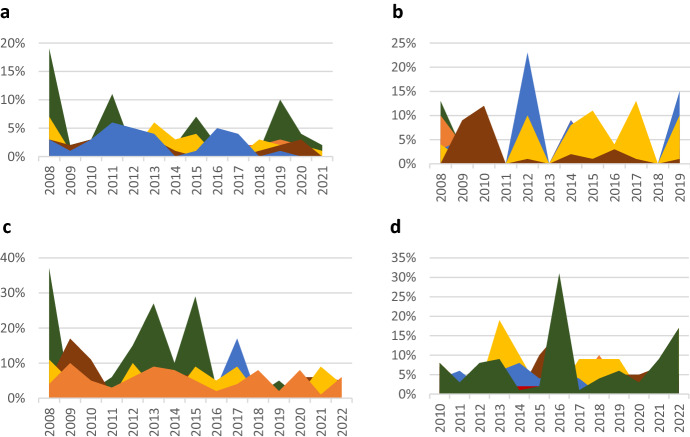
**a** Aggregated differences between General Secretary and other actors. **b** Aggregated differences between Premier and other actors. **c** Aggregated differences between the State Council Information Office and other actors. **d** Aggregated differences between the Foreign Minister and other actors

Pronounced discrepancies appeared between Chinese foreign policy actors, as Fig. 5 illustrates. The divergence between the CCP General Secretary/State President and other actors was the lowest. The degree of divergence between the State Council Information Office and other actors was highest, followed by the Foreign Minister. For *defend China’s territory, political system, and citizens,* discrepancies between Chinese foreign policy actors were particularly pronounced. Between the CCP General Secretary/State President’s and other actors’ statements, the most pronounced differences appeared in *control the region*. Between the Premier and other actors’ statements, pronounced differences first appeared in *offer global public goods*, then mainly regarding *lead global governance*. The State Council Information Office’s statements showed big discrepancies in *expand China’s economic relations* compared to statements from other actors. The analysis shows that the General CCP Secretary/State President disproportionately influences the construction of China’s national interest. Other foreign policy actors tend to follow his lead. However, noteworthy differing views regarding the importance of *defend China’s territory, political system, and citizens, control the region,* and *lead global governance* appeared between him and the other actors.

**Fig. 6:**
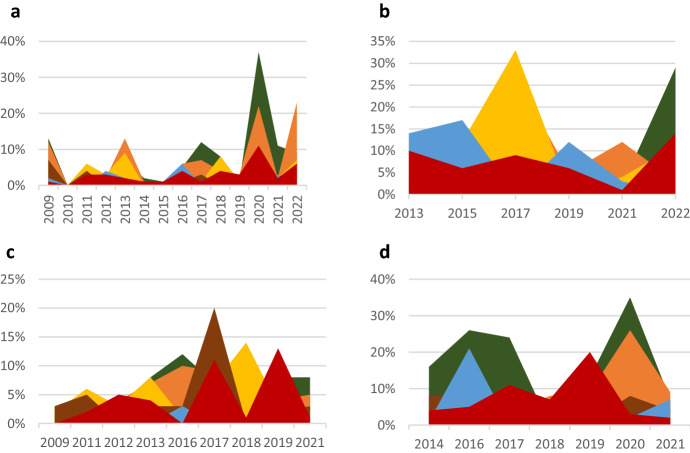
**a** Aggregated differences between the President and other actors. **b** Aggregated differences between the Vice-President and other actors. **c** Aggregated differences between the Secretary of State and other actors. **d** Aggregated differences between the Secretary of Defense and other actors

In the USA, the highest degree of divergence appeared between the Vice-President and other foreign policy actors. The smallest degree of divergence featured between the Secretary of State and other foreign policy actors. There were also big differences between the Secretary of Defense and other foreign policy actors, especially regarding promoting US values. After 2016, particularly after 2019, discrepancies between the US President and other foreign policy actors increased significantly. As Fig. 6a shows, around 2016, discrepancies covered all components of the construction of the national interest. Around 2019, discrepancies were most pronounced for *defend the country’s territory, political system, and citizens*, *expand its economic relations*, and *promote the country’s values*. In the USA, the State Department seems to take the lead, and other actors follow. Discrepancies between the US President and other actors appeared during the Trump administration, especially toward the end.

The analysis showed that different foreign policy actors put different degrees of emphasis on the different components of the construction of the national interest. The divergences between foreign policy actors reflect differing positions and substantial debate behind the formation of grand strategies. On the Chinese side, the divergence between foreign policy actors encompasses almost all components of the construction of China’s national interest, except *promote China’s values*. In the USA, the divergence between foreign policy actors centers around exactly these components of the construction of the national interest.

## Conclusion

Breaking down the construction of the US and China’s national interest into six components, this article found important differences in emphasis in the USA and China’s grand strategies and substantial divergence within both countries. Most importantly, the Chinese government seeks to play a more prominent role internationally, whereas the US government is increasingly concerned about the country’s security and economic standing. In many instances, the manifestations of US and Chinese grand strategies mirror each other. Most recently, the competition between political systems and associated values has been gaining traction, first on the Chinese, then on the US side. While there is more divergence between policy levels in the USA, even in the PRC’s highly centralized authoritarian system, there are still discrepancies between the strategic and policy planning level. In the USA, disagreements between foreign policy actors centered around promoting the country’s values internationally. In China, most differences appeared regarding territorial defense.

Two main limitations come with solely focusing on the rhetorical level of foreign policy. First, there might be big differences between what is publicly communicated and what is discussed behind closed doors. Second, there can be big gaps in the official portrayals of a country’s national interest and how it pursues it. This article cannot account for such gaps between foreign policy rhetoric and behavior. However, as presented in this article, longer-term systematic analyses of official foreign policy statements can serve as a jumping-off point for identifying such gaps. At the same time, it is still worth paying close attention to foreign policy rhetoric, especially if one analyses it carefully and finds discrepancies between different actors. Here, it is important to acknowledge the time-boundedness of the analysis, which limits the generalizability of its findings beyond the analyzed time frame.

This article showed that adopting a longer-term perspective and considering statements from different foreign policy actors within each state to assess great powers’ grand strategies is crucial. Bringing in the substance of the construction of the national interest on both sides gives indications about the underpinnings of Sino-US strategic competition. It shows how coherent the construction of the national interest is on both sides and where there might be clashes between the two sides. Future research should assess how and to what extent the Chinese and US governments follow the expressed priorities in their foreign policy behavior. In addition, it should find explanations for the uncovered discrepancies.

## Footnotes

1 The strategic level covers the Political Work Report to Party Congress (*n* = 2), the CCP General Secretary’s domestic (*n* = 8) and international (*n* = 79) speeches. The policy planning level covers the Government Work Report (*n* = 14), the Five-Year-Plan (*n* = 3), the Foreign Minister’s speech at the annual symposium for international affairs (*n* = 8), policy papers (*n* = 19), the Premier’s (*n* = 10), and the Foreign Minister’s (*n* = 16) international speeches.

2 The strategic level covers the National Security Strategy (*n* = 4), the State of the Union address (*n* = 13), other speeches by the President in front of domestic audiences (*n* = 14), and the President’s (*n* = 21), and Vice-President’s international speeches (*n* = 8), for example, at the United Nations, World Economic Forum, and the Munich Security Conference (*n* = 8). The policy planning level covers the Secretary of State’s domestic (*n* = 25) and international (*n* = 4) speeches as well as the Secretary of Defense’s domestic (*n* = 7) and international (*n* = 4) speeches.

3 A complete list of analyzed document will be made available on the author’s personal website.

4 The Chinese government publishes official English translations for most of its foreign policy statements. Previous research by Mokry (2022) suggests that there might be important differences between the English- and Chinese-language version of the same document. The author followed Mokry’s suggestion and systematically compared the original Chinese-language version of each document with its official English translation to check whether there were differences between the two versions that would affect the analysis. Since the identified differences between the Chinese- and English language versions were too small to affect the results of the quantitative content analysis, they do not need to be discussed in this article.

5 These findings coincide with expectations derived from power transitions theory.
